# Mechanobiological Feedback in Pulmonary Vascular Disease

**DOI:** 10.3389/fphys.2018.00951

**Published:** 2018-07-25

**Authors:** Paul B. Dieffenbach, Marcy Maracle, Daniel J. Tschumperlin, Laura E. Fredenburgh

**Affiliations:** ^1^Division of Pulmonary and Critical Care Medicine, Department of Medicine, Brigham and Women’s Hospital, Boston, MA, United States; ^2^Schulich School of Medicine and Dentistry, Western University, London, ON, Canada; ^3^Department of Physiology and Biomedical Engineering, Mayo Clinic College of Medicine and Science, Rochester, MN, United States

**Keywords:** vascular stiffness, pulmonary arterial stiffness, pulmonary hypertension, mechanotransduction, cellular mechanosensors, YAP/TAZ, matrix stiffness, vascular remodeling

## Abstract

Vascular stiffening in the pulmonary arterial bed is increasingly recognized as an early disease marker and contributor to right ventricular workload in pulmonary hypertension. Changes in pulmonary artery stiffness throughout the pulmonary vascular tree lead to physiologic alterations in pressure and flow characteristics that may contribute to disease progression. These findings have led to a greater focus on the potential contributions of extracellular matrix remodeling and mechanical signaling to pulmonary hypertension pathogenesis. Several recent studies have demonstrated that the cellular response to vascular stiffness includes upregulation of signaling pathways that precipitate further vascular remodeling, a process known as mechanobiological feedback. The extracellular matrix modifiers, mechanosensors, and mechanotransducers responsible for this process have become increasingly well-recognized. In this review, we discuss the impact of vascular stiffening on pulmonary hypertension morbidity and mortality, evidence in favor of mechanobiological feedback in pulmonary hypertension pathogenesis, and the major contributors to mechanical signaling in the pulmonary vasculature.

## Introduction

Pulmonary Hypertension is characterized by progressive pulmonary vascular remodeling that leads to exertional dyspnea, severe hypoxemia, and ultimately to right heart failure. The most severe form of the disease, PAH, initially and primarily affects the pulmonary arterial tree, and is associated with a 5-year survival of 61% (Farber et al., 2015). Other forms of PH result from other cardiac or pulmonary pathologies, but can progress to severe arterial remodeling (Rabinovitch et al., 1979; Wilkinson et al., 1988; Tuder et al., 2007; Kulik, 2014). For PH with significant vascular remodeling, treatment options are currently limited to pulmonary vasodilator therapies. These can lead to modest improvements in exercise capacity, but remain unable to reverse the disease process or significantly prolong survival (Fredenburgh et al., 2009; McLaughlin et al., 2009; Sutendra and Michelakis, 2014; Farber et al., 2015). These limitations have motivated extensive research to disrupt or reverse vascular remodeling.

Pulmonary arterial stiffness is increasingly appreciated as an important marker and predictor of disease severity and poor functional status in PH (Wang and Chesler, 2011; Schafer et al., 2016; Thenappan et al., 2016). Multiple lines of evidence indicate that increased vascular stiffness occurs prior to detectable changes in characteristic hemodynamic parameters, such as increased PVR (Pellegrini et al., 2014; Dragu et al., 2015; Ploegstra et al., 2018). Unlike in the systemic circulation, both proximal and distal vessels contribute to PA compliance, and current research has found evidence for early stiffening throughout the pulmonary vascular tree. Multiple processes may contribute to this vascular stiffening *in vivo*, including vascular smooth muscle tone, arrangement and structure of arterial laminae, local composition of extracellular matrix, and alterations in arterial geometry (thickness, diameter, and branching pattern).

This review will briefly address our current understanding of the clinical impact of pulmonary vascular stiffness. We will then focus on evidence for PA stiffening as a critical early driver of vascular remodeling – a process known as mechanobiological feedback. A close examination of the molecular mechanisms by which vascular cells promote, sense, and pathologically respond to stiffening is critical for our understanding of this process. Therapeutic targeting of key mechanotransduction mediators offers the potential to disrupt mechanobiological feedback and prevent or reverse pathologic vascular remodeling.

## Clinical Impact of Vascular Stiffening in PH

Increased vascular stiffness in the systemic circulation is strongly associated with incident systemic hypertension, and has been convincingly shown to precede the development of hypertension in multiple cohorts (Mitchell et al., 2004; Kaess et al., 2012; Weisbrod et al., 2013). Furthermore, arterial stiffness is a predictor of cardiovascular mortality (Boutouyrie et al., 2002; Wang et al., 2010; Ben-Shlomo et al., 2014). Loss of compliance in the large arteries results in increased pulse pressure, pulsatile afterload, and flow pulsatility, which have been shown to impact left ventricular (LV) remodeling (Kelly et al., 1992; Kaess et al., 2016; Bell et al., 2017) and promote microvascular damage in distal vascular beds of the brain and kidney (Hashimoto and Ito, 2011; Cooper and Mitchell, 2016; Pase et al., 2016; Tsao et al., 2016; Huang et al., 2017). Similarly, recent evidence from both patients and disease models has led to a growing appreciation for the impact of vascular stiffening in the pulmonary circulation as well (Wang and Chesler, 2011; Schafer et al., 2016; Thenappan et al., 2016).

Several groups have examined associations between mortality and non-invasive measures of proximal pulmonary vascular stiffness. Measurement techniques have varied across groups, and include intravascular ultrasound (Ploegstra et al., 2018) or MRI (Gan et al., 2007) to measure proximal arterial luminal area change (and calculate PA distensibility), as well as more global measures of vascular system capacitance [right ventricular (RV) stroke volume/pulse pressure] via cardiac catheterization or echocardiography (Mahapatra et al., 2006a,b; Pellegrini et al., 2014; Al-Naamani et al., 2015). These measurements of vascular stiffness have been strongly associated with mortality in pediatric PAH patients (Ploegstra et al., 2018), idiopathic PAH cohorts (Mahapatra et al., 2006a,b; Gan et al., 2007), scleroderma-associated PH (Campo et al., 2010), and PH in the setting of heart failure (Pellegrini et al., 2014; Al-Naamani et al., 2015; Dragu et al., 2015). Several studies demonstrated that stiffness measures were better predictors of mortality than the more typically measured resistance indices (PVR, PVR index, transpulmonary gradient) (Mahapatra et al., 2006b; Pellegrini et al., 2014; Al-Naamani et al., 2015; Dragu et al., 2015; Ploegstra et al., 2018), and particularly demonstrated prognostic utility in patients with normal PVR (Pellegrini et al., 2014; Dragu et al., 2015; Ploegstra et al., 2018).

Beyond its utility as a marker of mortality, PA stiffness may better predict early disease and functional status in PH than PVR and other traditional clinical parameters (Hunter et al., 2008; Sanz et al., 2009; Kang et al., 2011; Lau et al., 2016; Malhotra et al., 2016). One group measured vascular impedance (a global measurement of opposition to pulsatile flow that incorporates resistance and arterial stiffness) using pulse-wave Doppler ultrasound and right heart catheterization in a cohort of patients with PAH (Hunter et al., 2008). Impedance was a better predictor of worsening clinical status than PVR index alone in PAH patients, and particularly improved predictions of mild clinical worsening. Kang et al. (2011) used same-day MRI measurements with right heart catheterization to calculate proximal PA distensibility, and demonstrated that this was a better predictor of poor functional status (6MWD < 400 m) than PVR index or pulmonary capacitance in a small PAH cohort. Another set of investigators demonstrated that these MRI-derived measures of PA stiffness correlate well with hemodynamic severity and were sensitive to abnormal pulmonary vascular response to exercise that may represent early disease (Sanz et al., 2009). More recently, global PA distensibility was calculated during cardiopulmonary exercise testing using the Linehan distensible vessel model (Linehan et al., 1992; Malhotra et al., 2016). In a mixed cohort of patients with heart failure and PAH, arterial distensibility was a good independent predictor of peak oxygen consumption in a multivariable analysis that included resting hemodynamics (Malhotra et al., 2016). Lau et al. (2016) used a similar strategy to demonstrate decreased global arterial distensibility in patients with normal resting hemodynamics who had known (thromboembolic or biopsy-proven) PH or who subsequently developed PH during follow-up.

**FIGURE 1:**
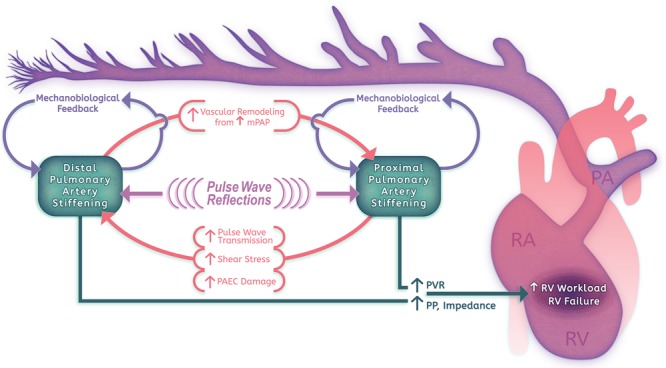
Pulmonary arterial stiffening in PAH pathogenesis.

The identification of early changes in PA stiffness and of strong associations between stiffness and PH morbidity has validated PA distensibility as a marker for early disease. This has motivated further investigation into whether vascular stiffening plays a key role in PH pathogenesis and progression (**Figure 1**). Physiologically, there is strong evidence that arterial stiffening contributes significantly to RV afterload and RV failure. Standard measurements of RV afterload using PVR account for only the steady-flow component of RV work, however, arterial stiffness is the critical determinate of RV pulsatile afterload (Tan W. et al., 2014; Bloodworth et al., 2015). In the pulmonary circulation, pulsatile afterload contributes approximately 23% to the workload of the RV, and has not been found to change through the course of disease (Saouti et al., 2010a). Unlike vascular resistance, which is localized primarily to small arteries and arterioles, pulsatile afterload reflects the status of vessels throughout the pulmonary vascular tree (Saouti et al., 2010a; Wang and Chesler, 2011). Proximal large artery stiffness has been comparatively well studied, but only contributes 15–25% of the total oscillatory work; direct measurement of the distal vasculature has been difficult to obtain (Bloodworth et al., 2015). Through disease modeling and advanced imaging approaches, measures of PA stiffness are increasingly being used to evaluate RV function and outcomes. These pioneering physiological studies suggest that evaluating PH without taking into account vascular stiffness is incomplete; incorporating stiffness measures allows a more complete analysis of RV workload, improves outcome prediction, and will likely be useful for monitoring response to therapy.

One important physiologic relationship that has emerged from evaluations of global pulmonary artery stiffness is that PVR and PA capacitance (a measure inversely related to stiffness) have an inverse hyperbolic relationship (Lankhaar et al., 2008; Saouti et al., 2009). In particular, Lankhaar et al. (2008) demonstrated that this relationship holds true in patients with and without PH and that current treatments do not greatly alter this coupling. Clinically, this inverse relationship means that large measurable changes in compliance are manifested by relatively small changes in resistance in early disease, and that large measurable changes in resistance yield only small changes in compliance in later disease (Lankhaar et al., 2008; Saouti et al., 2010b). These authors suggest that this physiology in large part explains why measures of vascular stiffness are excellent markers for early PH (Wang and Chesler, 2011; Thenappan et al., 2016).

Increasing evidence from disease models also implicates a critical role for PA stiffening in a positive feedback cycle of pathologic vascular remodeling. Distal vasculopathy can promote increased mean pressures, resulting in large vessel wall thickening, vessel dilation, and extensive alterations in ECM content (Rabinovitch et al., 1979; Birukova et al., 2013). Stiffening in the proximal vasculature can amplify pulse wave transmission to the distal vessels, resulting in shear stress, inflammation, and smooth muscle cell (SMC) remodeling behaviors (Li et al., 2009; Scott et al., 2013). Finally, vascular stiffening itself may promote local remodeling through alterations in gene expression and cellular behaviors in response to the local mechanical microenvironment – a process we have termed mechanobiological feedback (Bertero et al., 2015; Liu et al., 2016; Dieffenbach et al., 2017). We will discuss the impact of both proximal and distal PA stiffening on vascular remodeling in further detail below.

## Proximal PA Stiffening and Its Consequences on Vascular Remodeling

Stiffness of proximal pulmonary arteries is determined in large part by the composition and structure of the ECM proteins comprising large portions of the vessel wall. These proteins include proteoglycans and glycoproteins, but elastin and collagen predominate (Tsamis et al., 2013). Elastin is a resilient, flexible fiber that allows repetitive stretching of the vessel wall, whereas collagen provides strength but is much less deformable, limiting arterial compliance and preventing damage to the vessel (Tsamis et al., 2013; Schafer et al., 2016). As a result, in healthy tissues at low strains the more compliant mechanical properties of elastin predominate, with collagen carrying loads only under high-strain conditions (Hunter et al., 2011). Remodeling of these arteries during PH leads to vessel dilation, inflammatory cell accumulation, degradation of elastin, and collagen accumulation (Lammers et al., 2008). Examination of mice with variable vascular elastin expression shows a gradual increase in mPAP that inversely correlates with elastin levels (Shifren et al., 2008). Specifically, *ex vivo* biaxial strain testing and accompanying two-photon microscopy in explanted human tissues demonstrated severe elastin fragmentation and collagen accumulation in PH that correlated well with collagen-predominant (stiff) mechanics even under low-strain conditions (Rogers et al., 2013). Work in the chronic hypoxia neonatal calf model has also shown that the elastin layer can stiffen to allow adaptation to higher pressures, but this may primarily be a response observed early in life when elastin synthesis is present at much higher levels (Lammers et al., 2008; Schafer et al., 2016).

For many years, the standard dogma has been that proximal vessel stiffening is a relatively late disease manifestation, however, studies in animal models increasingly demonstrate that proximal stiffening occurs relatively early after exposure to pathologic stimulation (Tan W. et al., 2014). Seminal work by Myrick and Reid suggested early increases in proximal pulmonary artery stiffness in rats in the setting of hypoxia, with an increase in artery diameter of more than 50% after 3 days of exposure (Meyrick and Reid, 1979b). These investigations later showed a doubling in the thickness of the elastic lamina after 10 days of exposure, with collagen and elastin contributing proportionally (Meyrick and Reid, 1980). In the same rat hypoxia model, the Riley laboratory group found increased collagen synthesis by 3 days and a twofold increase in hydroxyproline wall content within 5 days of hypoxia exposure that correlated with a doubling in stiffness of the PA bed, as measured by pressure-volume loops (Poiani et al., 1990; Tozzi et al., 1994). Kobs et al. (2005) more recently performed *ex vivo* testing of the passive mechanical properties of mouse PAs in the hypoxia model at relatively early time points (1–2 weeks of hypoxia). They found a significant (∼25%) increase in effective elastic modulus and decreased compliance of proximal mouse PAs that correlated histologically with proportional thickening of both collagen and elastin elements of the arterial wall (Kobs et al., 2005).

Work by the Rabinovitch laboratory has demonstrated early internal elastic lamina disruption and increases in elastolytic activity in the monocrotaline (MCT) rat model of PH within the first few days after injection, and weeks before the development of hemodynamic changes (Todorovich-Hunter et al., 1992). Serine elastase appears to be responsible for this degradation, and its upregulation is associated with later development of neointimal lesions (Kim et al., 2011). Furthermore, disruption of this early change in stiffness via serine elastase inhibition is beneficial in preventing or reversing elevated PA pressures and RV hypertrophy. This has been convincingly shown in multiple disease models, including hypoxia, MCT, S100A4, and sugen-hypoxia models of PH (Maruyama et al., 1991; Ye and Rabinovitch, 1991; Cowan et al., 2000a; Zaidi et al., 2002; Kim et al., 2011; Nickel et al., 2015), although recent studies suggest an independent effect on bone morphogenic protein signaling that may also contribute (Nickel et al., 2015). Collagen remodeling has also been found to be important in this early time period, as mice with impaired ability to degrade collagen fail to improve proximal vascular stiffening and tend to have higher PA pressures during recovery (Ooi et al., 2010).

A large body of recent work by Stenmark, Tan, and colleagues has elucidated a putative mechanism by which early proximal pulmonary vascular stiffening can impact PH progression via induction of high pulsatile flow in the distal vasculature (Li et al., 2009, 2013; Scott et al., 2013; Tan W. et al., 2014; Tan Y. et al., 2014). In the pulmonary circulation, proximal pulmonary arteries are 10–20-fold more compliant compared to systemic arteries (Lammers et al., 2008), but smaller vessels also contribute significantly (∼80%) to the total compliance of the pulmonary vascular tree (Saouti et al., 2010b). Nevertheless, the large and medium-sized elastic pulmonary arteries of the upstream vasculature expand passively during systole and recoil during diastole, serving to convert pulsatile flow to a more constant flow in distal vessels while also dampening pressure variations throughout the cardiac cycle (Tan W. et al., 2014). Constant, low-pulsatility laminar flow is physiologic for vascular endothelium, resulting in high levels of NO production, endothelial barrier integrity, and low expression of adhesion and procoagulatory molecules. Experimental disturbance of flows from this state *in vivo* lead to compensatory vascular remodeling that functions to maintain shear stresses near physiologic levels, but may also contribute to pathological processes such as atherosclerosis (Chiu and Chien, 2011). During PH progression, stiffer proximal vessels will distend less with each stroke volume and therefore transmit greater flow and pressure pulsatility to the more distal vasculature (Scott et al., 2013; Su et al., 2013).

To study the effects of increased flow transmission from stiffened proximal arteries, these investigators used an *in vitro* system that simulated high flow pulsatility compared to low flow pulsatility with the same mean flow stresses (simulating the same static pressures/mPAP) on human PAECs. Compared to normal flow pulsatility, high flow pulsatility led to attenuated release of the pulmonary vasodilators NO and prostaglandin-F1α (PGF1α) and enhanced release of the potent vasoconstrictor endothelin (Li et al., 2009). Over short timeframes, this change in pulsatility induced expression of inflammatory leukocyte adhesion molecules (such as intercellular adhesion molecule, endothelial leukocyte adhesion molecule, and monocyte chemo-attractant protein-1) and led to endothelial cell proliferation (Li et al., 2013). Using a co-culture system of PAECs and PASMCs, these authors demonstrated that high-pulsatility flow induced PASMC hypertrophic responses, including expression of the contractile proteins α-smooth muscle actin (αSMA) and smooth muscle myosin heavy chain, without affecting proliferation (Scott et al., 2013). Treatment with a variety of vasodilators, including ACE inhibitors and endothelin antagonists, could prevent this effect. The putative mechanosensing mechanisms underlying endothelial responses to shear stress will be discussed below. These *in vitro* models suggest that proximal vascular stiffening may induce or exacerbate distal vascular vasoconstriction, inflammation, and remodeling, providing a key contribution to the progression of disease.

## Distal and Microvascular PA Stiffening and Mechanobiological Feedback

Muscularization of normally non-muscularized “intra-acinar” pulmonary arteries was recognized as an early finding (day 3 of exposure) by Meyrick and Reid in their initial studies of hypoxia- and Crotolaria-induced PH in rats (Meyrick and Reid, 1979a,b). This neomuscularization notably occurs before proliferation, and was found to represent hypertrophy and metaplasia of precursor SMCs not normally visible by light microscopy (Hislop and Reid, 1976; Meyrick et al., 1978). Recent lineage tracing experiments by the Greif lab in the mouse hypoxia model have meticulously identified these SMC progenitors, which arise from the border region between muscular and non-muscular arteries and express both αSMA and the undifferentiated mesenchymal marker platelet derived growth factor receptor-β (Sheikh et al., 2014, 2015). Early in the development of hypoxia-induced PH, individual progenitor cells migrate distally, dedifferentiate, and clonally expand to enable muscularization in the distal arteries by days 5–10 post-hypoxia (Sheikh et al., 2015).

Our recent work has added to the field by directly characterizing the stiffness of the pulmonary vasculature locally at the micron scale in models of PH and human PAH. To do so, we developed a methodology to characterize the local elastic properties of the lung using atomic force microscopy (AFM) (Liu F. et al., 2010; Liu et al., 2015; Liu and Tschumperlin, 2011). Optimal measurements use large spherical tips (1–2.5 μm radius) on thick (20–50 μm) sections with sufficient force (50 pN) to interrogate vessel mechanical properties (Sicard et al., 2017). Although this method requires unfixed tissue, limiting some applications, it allows for unparalleled spatial resolution to measure local tissue stiffening in even the smallest vessels (Liu and Tschumperlin, 2011). Examination of human tissue has allowed us to determine the range of medial stiffness relevant to disease (Liu et al., 2016), and interestingly demonstrated a normal ∼2-fold increase in PA stiffness with aging (Sicard et al., 2018). These observations raise the possibility that some forms of PH may be related to an accelerated aging phenotype or represent genetic/epigenetic dysregulation of the normal age-related process.

Using this AFM approach, we examined the spatial and temporal distribution of PA stiffening in both the MCT and sugen-hypoxia rat models of PH (Liu et al., 2016). We found that there is an early (7 day) 1.5–2-fold increase in pulmonary artery stiffening in distal vessels (<100 μm in diameter) in both the MCT and sugen-hypoxia models that subsequently progresses to an approximately sixfold (0.6 kPa to 3.6–4 kPa) increase in vascular stiffness in later stage disease (Liu et al., 2016). Wall thickening detectable by light microscopy and more proximal arterial medial stiffening (vessel diameter 100–300 μm) occurs subsequent to this distal stiffening, followed by changes in PA pressures and frank RVH. Taken together, our results suggest that medial PA stiffening arises early in distal vessels and progresses proximally in these models, preceding detectable hemodynamic changes and frank RV dysfunction. Notably, our methodology examines local-scale changes in stiffness in the vessel media of intrapulmonary vessels, so we would not have detected early changes in elastic laminae of the large arteries discussed above. Current evidence indicates that both proximal and distal vascular remodeling are likely important to disease pathogenesis, but their relative contributions and the direction of propagation require further study, particularly in human disease.

These changes in the local mechanical environment in early PH suggest a potential role for PA stiffness in the progression of disease. From a physiologic standpoint, smaller vessels contribute significantly to total arterial compliance as well as to vascular resistance; thus, increased stiffness and muscularization will impact both static and oscillatory RV workload (Saouti et al., 2010b). Due to pulse-wave reflection and proximal artery dilation in response to mean pressure changes, there will be increased circumferential stress on proximal vessels, contributing to inflammation and vascular remodeling in these vessels (**Figure 1**; Wang and Chesler, 2011).

Beyond physiologic principles, the mechanical micro environment is being increasingly recognized for its critical role in regulating key cellular processes during organogenesis (Mammoto and Ingber, 2010) and in the development of pathology (Georges et al., 2007; Klein et al., 2009; Liu F. et al., 2010; Liu et al., 2015, 2016; Gulino-Debrac, 2013; Perepelyuk et al., 2013; Pickup et al., 2014; Huveneers et al., 2015; Tschumperlin, 2015). Pathogenic vascular stiffness has been found to precede the development of systemic hypertension (Kaess et al., 2012), and leads to microvascular endothelial and SMC dysfunction (Huveneers et al., 2015). We have extensively examined the response of pulmonary vascular cells to pathologic matrix stiffness *in vitro* using discrete stiffness polyacrylamide hydrogels (Liu F. et al., 2010; Liu et al., 2016; Dieffenbach et al., 2017), and have found that stiffness induces changes that can drive further vascular remodeling – a process we call mechanobiological feedback.

**FIGURE 2:**
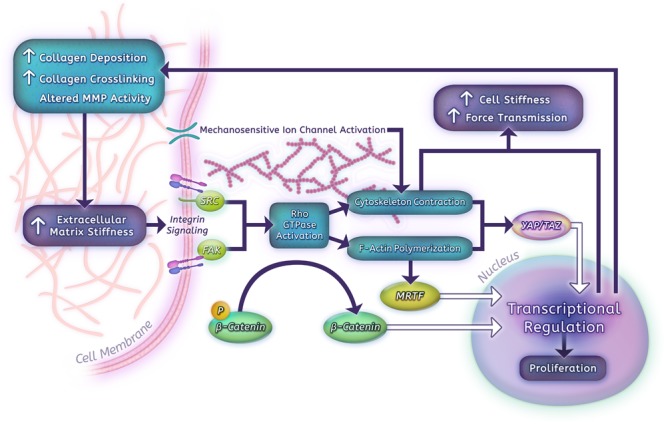
Mechanisms of mechanobiological feedback.

Human PASMCs and PAECs grown on hydrogels that span the range from normal parenchyma to highly remodeled vessels (shear modulus 0.1–25.6 kPa) show a robust proliferative response to matrix stiffness (Liu et al., 2016). In PASMCs, contractile force generation, as assessed by traction force microscopy, is likewise highly stiffness dependent (Dieffenbach et al., 2017), which may in part be due to remodeling of the actin cytoskeleton. These remodeling phenotypes correlate with increased expression of matrix proteins, such as collagen I, fibronectin, and LOX, a key collagen cross-linking enzyme (Liu et al., 2016; Dieffenbach et al., 2017). Additional downstream consequences of arterial stiffness include increased fibrotic changes and ECM modifications via a miR-130/301-mediated mechanism and stiffness-dependent alterations in glutaminolysis that impact proliferation and migration of vascular cells (Bertero et al., 2015, 2016). We have also found that stiffness-induced activation of pulmonary vascular cells is at least in part dependent on downregulation of cyclooxygenase-2 (COX-2) expression and COX-2-dependent prostanoid production (Fredenburgh et al., 2008; Liu F. et al., 2010; Liu et al., 2016; Dieffenbach et al., 2017). Importantly, suppression of matrix stiffening or stiffness-induced signaling using a LOX inhibitor (β-aminopropionitrile) or a long-acting prostanoid (iloprost) at the time of early PA stiffening prevented distal PA stiffening and later hemodynamic consequences of PH *in vivo*, demonstrating the therapeutic potential of disrupting mechanobiological feedback (Bertero et al., 2015; Liu et al., 2016). Over the past several years, a number of molecules have emerged as likely mediators of mechanobiologic feedback in the pulmonary vascular system, and may make promising future therapeutic targets to disrupt PH development and progression (**Figure 2**).

## Key Molecular Pathways Involved in Mechanobiological Feedback

### Non-structural Determinants of Matrix Stiffness

Vascular stiffness ultimately arises from a combination of factors, including increased ECM deposition, matrix stiffening, vascular cell proliferation/hypertrophy, and active smooth muscle contraction. The major structural ECM components and their role in large vessel stiffness were discussed previously, however, matrix composition and mechanical properties are also altered by enzymatic activity that is closely regulated at the cellular level (**Figure 2**).

As detailed above, serine elastase production is elevated in several models of PH (Maruyama et al., 1991; Ye and Rabinovitch, 1991; Cowan et al., 2000a; Zaidi et al., 2002; Kim et al., 2011; Nickel et al., 2015), and its expression has been found to correlate with development of neointimal lesions in one model (Kim et al., 2011). In addition to shifting workload to less compliant collagen fibers (Schafer et al., 2016), elastin fragmentation within the medial layer can directly promote a variety of SMC remodeling behaviors, including phenotype switching, migration, and proliferation (Chelladurai et al., 2012). Mechanistically, this occurs through liberation of matrix bound SMC mitogens such as fibroblast growth factor (Thompson and Rabinovitch, 1996) and exposure of fibronectin-derived peptides and previously hidden integrin binding sites (Kafienah et al., 1998). Inhibition of serine elastases can lead to regression of progressive medial PA hypertrophy in culture *ex vivo* (Cowan et al., 2000b), and is also associated with decreased PA remodeling in disease models (Maruyama et al., 1991; Ye and Rabinovitch, 1991; Cowan et al., 2000a; Zaidi et al., 2002; Kim et al., 2011; Nickel et al., 2015). Recent studies by the Stenmark and Harrison groups have also shown inflammatory activation of macrophages by elastin fragments (Schafer et al., 2016). Pidkovka and colleagues, in particular, reported upregulation of the elastase matrix metalloproteinase 12 (MMP12) by mechanical stretch *in vitro* and MMP12-dependent aortic macrophage accumulation and subsequent vascular stiffening *in vivo* (Luft, 2012).

MMP12 is one of a large family of MMPs and their endogenous inhibitors (tissue inhibitors of MMPs, or TIMPs) that likely play a role in altering the vascular mechanical microenvironment. MMP2 and MMP9 are metalloproteinases with diverse ECM cleavage functions which were initially identified by their ability to degrade collagen IV of the basement membrane; they are also referred to as gelatinases based on their efficient cleavage of denatured collagen I. Elevated MMP2 expression and collagen IV degradation are seen in PH neointimal lesions (Matsui et al., 2002), and gelatinase levels are elevated in both serum and PASMCs derived from patients with PH (Benisty et al., 2005; Cantini-Salignac et al., 2006; Chelladurai et al., 2012). MMP2 and MMP9 activity are also increased in MCT and hypoxia-induced PH models (Frisdal et al., 2001; Schermuly et al., 2004; George and D’Armiento, 2011). Disruption and turnover of the basement membrane, like the internal elastic lamina, is therefore likely important to the development of intimal and medial hypertrophy. Indeed, gelatinase inhibition can disrupt PA medial hypertrophy *ex vivo* (Cowan et al., 2000b). Much remains to be understood about the drivers and mechanical impact of these basement membrane alterations.

MMPs also have extensive non-matrix secondary effects via cytokine processing and release from the ECM that likely contribute to the complexity of their regulation and their role in disease (Chelladurai et al., 2012). On a more global level, changes in matrix altering activity through MMP inhibition have been shown to have a mixed phenotype in PH (Chelladurai et al., 2012). While adenoviral-induced overexpression of TIMP1 in the lung is protective in the pro-inflammatory MCT PH model (Vieillard-Baron et al., 2003), both viral-mediated TIMP1 induction and MMP inhibition with doxycycline lead to increased vascular remodeling in the setting of chronic hypoxia (Vieillard-Baron et al., 2000). It is likely that initial collagen breakdown and ECM turnover contribute to PASMC proliferation and vascular remodeling through growth factor release and mechanotransduction, followed by increased collagen deposition and vascular stiffening (Bloodworth et al., 2015). Further study is greatly needed to clarify this process.

Lysyl oxidases are copper-dependent amine oxidase enzymes responsible for covalent cross-linking of collagen and elastin fibers, a process that imparts structural stability. Recently, Nave et al. (2014) identified upregulation of LOX family members (LOX, LOXL1, LOXL2, LOXL3, LOXL4) in multiple forms of human PH, including within plexiform and concentric vascular lesions. Expression of LOX, LOXL1, and LOXL2 was upregulated in human PASMCs in response to hypoxia, and increased in both the MCT rat model and the hypoxia mouse model of PH. In the hypoxia model, inhibition of LOX attenuated small artery muscularization, reduced vascular wall thickness and elastin accumulation, and decreased total collagen accumulation and cross-linking (Nave et al., 2014). We and the Chan laboratory have expanded on these findings, demonstrating that stiff matrix could induce LOX expression in PAAFs, as well as increase fibrillar collagen isoforms and collagen expression (Bertero et al., 2015). In particular, LOX upregulation was a key component of a complex mechanobiological positive feedback loop along with mechanotransductors YAP/TAZ and MiR 130/301 (see below) that led to progressive ECM stiffening *in vivo*. We verified the impact of LOX inhibition on hypoxia-induced PH, and tied it directly to inhibition of this mechanotransduction circuit. More importantly, we closely examined the impact of LOX inhibition in the MCT PH model, and demonstrated decreased collagen deposition and remodeling, reduced medial thickening, and directly decreased arterial stiffness as assessed by AFM (Bertero et al., 2015). These findings correlated with improvement in RV systolic pressures and RVH, as well as reduced activity of downstream mechanotransduction effectors as indicated above. The impressive impact of LOX inhibition in the MCT and hypoxia models illustrates successful disruption of mechanobiological feedback, and serves as a useful demonstration of the potential power of this approach.

Transglutaminase-2 (TG2) is a calcium-dependent crosslinking enzyme that is known to remodel collagen I and other components of the ECM, resulting in increased matrix stability and tissue rigidity (Eckert et al., 2014). In the systemic circulation, abolition of endothelial NO synthase (eNOS) activity upregulated matrix TG2 expression and crosslinking, and led to increased aortic and carotid stiffening (Jung et al., 2013). In PH, the Fanburg laboratory has shown that TG2 activity is upregulated in multiple rodent disease models, as well as in the serum of patients with PAH (Wei et al., 2012; DiRaimondo et al., 2014). In human PASMCs, TG2 was regulated both by mechanosensitive calcium channels (TRPV4, see below) and by hypoxia-inducible factor 1α, and was required for hypoxia-induced PASMC hyperproliferation (Penumatsa et al., 2014). Finally, TG2 inhibitor studies in the mouse sugen-hypoxia model demonstrated TG2-dependent upregulation of fibrotic markers in the lung and RV (Penumatsa et al., 2017), and also showed attenuated PA pressure elevations with TG2 inhibitor treatment (DiRaimondo et al., 2014). These studies indicate TG2-mediated ECM crosslinking also contributes to matrix stiffness in PH development.

### Mechanosensors

All adherent cells derive critical signals from their interactions with the ECM and their mechanical microenvironment in order to regulate cell shape, survival, proliferation, and other phenotypes. Mechanical disruption can be converted to chemical signaling directly via mechanosensitive ion channels. In endothelial cells, a number of mechanosensitive channels, including the TRPV4 and PIEZO1 calcium channels and the inwardly rectifying potassium (Kir) channels, are responsible for shear-flow induced vasodilation through activation of eNOS (Hartmannsgruber et al., 2007; Mendoza et al., 2010; Wang et al., 2016; Ahn et al., 2017). Although not normally subject to blood shear flow, PASMCs are exposed to transmural interstitial flows proportionally to transmural pressure differences, and can also have direct exposure to luminal shear flow in the setting of PH-induced endothelial injury (Makino et al., 2011; Shi and Tarbell, 2011). Calcium signaling subsequently leads to pulmonary vasoconstriction (via activation of myosin light chain kinase) and proliferation (through activation of calmodulin kinase and pro-growth transcription factors) (Kuhr et al., 2012). Song et al. (2014) found that three known mechanosensitive ion channels, TRPV4, TRPM7, and TRPC6 were significantly upregulated in idiopathic PAH PASMCs compared to cells from non-diseased controls. This upregulation correlated with greatly increased cytoplasmic calcium in response to shear stress that could be partially blocked with TRPM7 and TRPV4 channel inhibitors. Yang X.R. et al. (2012) found that TRPV4 was upregulated in the setting of hypoxia in rats, and correlated with increased vascular tone in endothelium-denuded small PAs in this model. Furthermore, TRPV4 knockout mice demonstrated delayed and attenuated vascular remodeling in response to hypoxia, further supporting that these channels play a role in disease pathogenesis (Yang X.R. et al., 2012).

The transmission of mechanical information from the ECM to the cell cytoskeleton and signal transduction machinery is mediated primarily through integrin-based adhesions (Tschumperlin, 2011; Tschumperlin et al., 2018). Integrins, comprising heterodimers of α and β subunits, are transmembrane proteins that recognize specific matrix polypeptides, such as the arginine-glycine-aspartic acid (RGD) sequence in fibronectin. Integrin subunits have only small cytoplasmic domains, but serve as scaffolds for recruiting a variety of cell-signaling machinery into adhesion complexes. Activated integrins are connected to the actomyosin system structurally through interactions with linking proteins such as talin and vinculin (Sun et al., 2016). This machinery, often referred to as a “molecular clutch” allows cells to both transmit forces to the ECM and translate forces into molecular signaling (Case and Waterman, 2015). The inner workings of this machinery are just beginning to be understood. For example, some specific integrin heterodimers display catch-bond properties, allowing longer binding time under increased loading (Kong et al., 2009). Recently, Elosegui-Artola et al. (2016) demonstrated that the force-regulated unfolding of talin competes with integrin unbinding to generate a “rigidity threshold” for downstream mechanical activation in response to force transmission. In the setting of higher rigidity (above 5 kPa in their system), talin unfolding tends to occur before catch-bond release, exposing vinculin binding sites, activating adhesion complexes, and stimulating mechanotransduction signals (YAP nuclear translocation, see below). These processes likely occur through multiple mechanisms, including recruitment of additional integrins by talin and reinforcement of the mechanical clutch by vinculin-actin binding (Saltel et al., 2009; Hirata et al., 2014; Elosegui-Artola et al., 2016). This fine-tuned machinery allows for assembly of cell-matrix adhesion complexes that can respond rapidly to changes in applied forces (Tschumperlin et al., 2018).

As some of the most important mechanosensors, integrins likely have an extensive impact on mechanical signaling during the development of PH. In PASMCs, integrins are differentially regulated in PH disease models, with augmentation of downstream signaling (Umesh et al., 2011). In particular, α_1_, α_v_, and α_8_ signaling are increased in both MCT and chronic hypoxia, whereas α_5_ and β_1_ are decreased in chronic hypoxia and α_5_ and β_3_ are decreased in the MCT model (Umesh et al., 2011). Chronic vasoconstriction in the setting of worsening disease causes an integrin-dependent reorganization of the actin cytoskeleton favoring increased integrin contacts and cellular stiffening, and allows for an adaptive reduced engagement of the active contractile apparatus (Martinez-Lemus et al., 2004; Sehgel et al., 2013). In endothelial cells, the cytoskeleton is dominated by F-actin stress fibers that attach to integrin-based focal adhesions, and this organization is highly affected by vascular stiffness (Sun et al., 2016). Integrin signaling is involved in and enhances mechanoactivation of ion channels (Huveneers et al., 2015). TRPV4 and integrins play a synergistic role in a positive feedback loop driving endothelial cell alignment in response to cyclic stretch on flexible substrates (Thodeti et al., 2009). Integrin signaling is also known to drive extracellular transforming growth factor-β (TGFβ) activation through mechanically mediated release from its matrix-bound latent complex (Buscemi et al., 2011; Shi et al., 2011). Although high pulsatile flow has been found to increase TGFβ signaling (Scott et al., 2013) and altered TGFβ superfamily signaling is one of the hallmarks of familial and idiopathic PAH (Eickelberg and Morty, 2007; Aschner and Downey, 2016), direct mechanical activation of TGFβ has not yet been well studied in pulmonary vascular cells.

Cells also derive information regarding their mechanical microenvironment through cell-cell contact in the form of intercellular adhesions. The most studied of these cell-cell mechanotransducers are cadherin-based adhesions formed by membrane-spanning proteins of the cadherin family on adjacent cells (Tschumperlin et al., 2018). Several elegant experiments examining cell shape and cytoskeletal responses in cell monolayers have demonstrated conclusively that stiffening of the ECM, as well as internal or external tension forces, lead to remodeling of cell-cell junctions, changes in cell shape, and altered cell migration (Liu Z. et al., 2010; Krishnan et al., 2011; Huveneers et al., 2012). The increase in endothelial contractility and traction force development in stiff matrix via these mechanisms leads to enhanced responsiveness to angiogenic or inflammatory permeability factors during PH progression (Liu Z. et al., 2010; Huynh et al., 2011; Krishnan et al., 2011). In addition to investigations of effects of vascular stiffness, the mechanism of cell-cell response to luminal shear flow has been particularly well-studied in endothelial cells. Acute onset of shear stress or mechanical force leads to engagement of platelet endothelial cell adhesion molecule-1 (PECAM-1), along with vascular endothelial cadherin as an adaptor protein, leading to activation of downstream mechanotransductors (in particular Src family kinases) and upregulation of phosphoinositide 3-kinase and eNOS to regulate vascular tone (Osawa et al., 1997; Tzima et al., 2005; Conway et al., 2013). Fluid shear stress-mediated activation of PECAM-1 also triggers cytoskeletal remodeling in endothelial cells via interaction with integrins and downstream Rho GTPases (Tzima et al., 2001; Goldfinger et al., 2008; Collins et al., 2012). Cell stretch also can activate endothelial proliferation in a VE-cadherin-dependent fashion, likely via the downstream mechanotransductors YAP and TAZ (Neto et al., 2018). Thus, cell-cell adhesions contribute extensively to mechanical and biochemical signaling in ways that are highly relevant to endothelial homeostasis and endothelial injury.

### Notable Cytosolic Mechanotransducers

Downstream of cell-matrix and cell-cell adhesion molecules, a large network of biochemical and cytoskeletal-interacting pathways contribute to the transduction of mechanical signals, however, a few key molecules have emerged as critical (**Figure 2**). For instance, the Src family of tyrosine kinases and FAK are both recruited to focal adhesions, modulate integrin-cytoskeletal links, and regulate each other’s activity (Tschumperlin, 2011; Bloodworth et al., 2015). In particular, Src activity has been shown to increase FAK expression at the cell surface and lead to greater force transmission in vascular smooth muscle; conversely, inhibition of FAK decreases Src activity in PASMCs (Paulin et al., 2014; Bloodworth et al., 2015).

Interestingly, Src activity is elevated in rodent models of PH, and tyrosine kinases with strong Src family inhibitory properties demonstrate improved reversal of experimental PH with treatment (Pullamsetti et al., 2012). Src phosphorylation of p130Cas, a stretch-responsive protein that exposes Src substrate domains under tension, has been shown to mediate mechanotransduction via multiple partners (Zamir and Geiger, 2001; Sawada et al., 2006). Furthermore, p130Cas has been found to be elevated in the serum, distal pulmonary arteries, and cultured PAECs and PASMCs from PAH patients, as well as in hypoxia and MCT models, and serves to amplify pro-remodeling receptor tyrosine kinase activity in the setting of progressive disease (Tu et al., 2012).

Similar to Src, FAK localizes to focal adhesions, self-activates tyrosine phosphorylation, and interacts with numerous components in response to mechanical strain (Fluck et al., 1999; Lee et al., 2000; Wang J.G. et al., 2001; Provenzano et al., 2009). Studies with FAK inhibitors have shown that FAK activation is required for mechanosensing in migration of both fibroblasts (Wang H.B. et al., 2001) and PASMCS (Paulin et al., 2014), and that FAK inhibition can abrogate PH development in the MCT rat model (Paulin et al., 2014).

Mechanical cues downstream of integrin signaling can influence the regulation of small Rho GTPases, including RhoA, Rac1, and CDC42, to alter the dynamics of the actin cytoskeleton and influence cell migration and proliferation. Rho GTPases are activated through recruitment of guanine nucleotide exchange factors (Birukova et al., 2006). Although these factors are often recruited via calcium signaling (Pardo-Pastor et al., 2018) and/or activation of FAK and Src family kinases (Guilluy et al., 2011; Bae et al., 2014; Pardo-Pastor et al., 2018), the exact regulatory mechanisms are integrin and cell-context dependent, and in many cases have not been fully worked out (Hoon et al., 2016). Activated Rho GTPases all promote actin assembly. RhoA is more involved in stress fiber formation, and Rac1 and CDC42 in filopodia and lamellipodia formation, respectively (Hoon et al., 2016).

RhoA is required for focal adhesion assembly and operates within focal adhesion complexes to regulate the dynamics and contractile activity of the actin cytoskeleton (Ridley and Hall, 1992). RhoA and its best-known downstream effector, RhoA-associated protein kinase (ROCK), are stimulated by cell stiffness (Klein et al., 2009), and also contribute to increased intracellular stiffness through stress fiber development and stimulation of actomyosin contraction (Maekawa et al., 1999; Riento and Ridley, 2003; Wang et al., 2009). RhoA and ROCK have been studied extensively in cardiovascular diseases, and have been found to be involved in the development of PH in disease models and in human disease. In endothelial cells, ROCK activity downregulates eNOS (Takemoto et al., 2002), upregulates inflammatory markers, and is responsible for cytoskeletal responses to shear stress (Li et al., 1999; Tzima et al., 2001; Katoh et al., 2008; Conway et al., 2013). ROCK activity is increased in both the media and the intima of pulmonary arteries (Shimizu et al., 2013) and also in cultured PASMCs derived from patients with idiopathic PAH (Guilluy et al., 2009). Treatment with the ROCK inhibitor fasudil suppresses both MCT and hypoxia-induced PH in rodents (Abe et al., 2004, 2006). In PH patients, fasudil has been found to decrease PVR after short-term use, and has had modest success in early clinical trials (Zhang and Wu, 2017). Furthermore, vascular smooth muscle-specific ROCK2 knockout mice were protected from hypoxia-induced PH, decreasing the likelihood of any off-target explanation for fasudil’s success (Shimizu et al., 2013).

Proteomics-based efforts have assiduously identified more than 500 additional individual proteins that are associated closely with cell-matrix adhesion complexes and facilitate cellular interactions with the surrounding mechanical environment (Kuo et al., 2011; Schiller et al., 2011). The overwhelming complexity of the interactions that occur due to mechanical perturbations has led to greater focus on pathways that integrate this information to drive transcriptional programs in response to environmental changes (Tschumperlin et al., 2018).

### Transcriptional Mechanotransducers

Gene activation in response to mechanical signaling requires conveying mechanical information to the cell nucleus. While many transcription factors can play a role in this process, a small number of central coordinators of mechanical signaling appear to convey critical mechanical signals via nuclear translocation (**Figure 2**). β-catenin is a well-known transcriptional coactivator that is normally targeted for degradation in the cytosol via phosphorylation by glycogen synthase kinase-3β (GSK3β), the core member of the β-catenin destruction complex. β-catenin activation canonically occurs via wingless/integrase (Wnt) signaling pathways, and leads to β-catenin-dependent activation of processes involved in proliferation, survival, and migration in many cell types. In epithelial cells, mechanical stretch and tissue stiffness lead to activation of β-catenin downstream of RhoA and ROCK activation (Heise et al., 2011; Samuel et al., 2011). Although stiffness-induced activation has not been well studied in PH, enhanced canonical (β-catenin-activating) Wnt signaling was associated with multiple cell types derived from PAH patients in a large non-biased screen examining gene expression signatures associated with PAH (West et al., 2014). Enhanced Wnt signaling was also found in a separate screen for targets of miRNAs elevated in end-stage PAH (Wu et al., 2016). Pulmonary artery resistance vessels from patients with idiopathic PAH demonstrated endothelial upregulation of β-catenin in idiopathic PAH vessels that correlated with upregulation of RhoA, ROCK, and Rac1 as assessed by immunohistochemistry and qPCR (Laumanns et al., 2009). Finally, downregulation of β-catenin reduces hypoxia-induced PASMC proliferation and PA remodeling in hypoxia models of PH (Yu et al., 2013; Alapati et al., 2014; Jin et al., 2015). These data together indicate a potential role for β-catenin activation downstream of stiffness-induced vascular remodeling in PH.

Nuclear factor-kappa B (NFκB) is a transcription factor most well-known for its involvement in inflammatory and immune responses, including flow-mediated inflammatory responses in endothelial cells (Davis et al., 2004; Orr et al., 2008; Petzold et al., 2009). The Stenmark laboratory group have shown that NFκB activation and subsequent upregulation of inflammatory markers occurs early and continuously in the setting of high-pulsatility flow (Li et al., 2013). Furthermore, this activation is dependent upon cell polarity changes and structural reorganization via actin and microtubule remodeling (Li et al., 2013). NFκB activation is also known to be sensitive to calcium influx, and can be activated by mechanical stretch-induced calcium currents via the TRPC3 channel in fibroblasts (Ishise et al., 2015). In endothelial cells, non-receptor activated currents through TRPC3 can lead to NFκB activation and expression of inflammatory cell adhesion molecules (Smedlund et al., 2010). Inhibition of NFκB genetically in the lung reduced endothelial apoptosis, endothelial-mesenchymal transition, and development of PH in the MCT model. Although further study on mechanical signaling via NFκB is needed, the current studies indicate a role for NFκB in inflammatory and endothelial cell responses to alterations in the mechanical microenvironment.

One critical link between stiffness-mediated cytoskeletal remodeling and gene expression are the MRTFs. The MRTFs form stable complexes with monomeric soluble actin (G-actin), leading to the sequestration of these complexes in the cytoplasm. Polymerization of G-actin into F-actin filaments and stress fibers in the setting of increased mechanical stress or migration liberates these factors to translocate to the nucleus, where they act as cofactors for serum response factor (Tschumperlin et al., 2018). Activity of MRTFs regulates the expression of multiple gene products central to contractile machinery in myofibroblasts (Small et al., 2010; Huang et al., 2012; Haak et al., 2014; Scharenberg et al., 2014), and is a downstream effector of profibrotic signaling by matrix stiffness (Huang et al., 2012) and Rho/ROCK pathways in this setting (Esnault et al., 2014). As stiffness-driven activators of pro-fibrotic and contractile activity, MRTFs are therefore critical components of a profibrotic mechanobiological feedback loop. In endothelial cells, MRTF-A has been shown to mediate inflammatory marker production downstream of RhoA activity in response to exposure to oxidized LDL (Fang et al., 2011). In vascular smooth muscle, MRTF-A was likewise involved in inflammatory mediator activation downstream of endothelin-1 activity (Yang Y. et al., 2014). Inhibition of MRTF-A in the pulmonary vasculature using shRNA injection significantly attenuated the development of hypoxic PH in rats (Yuan et al., 2014). This improvement correlated with reduced expression of inflammatory chemokines in the lung, ECM protein production by PASMCs, and collagen deposition in the PA beds (Yuan et al., 2014). Whether MRTF contributes significantly to mechanobiological feedback in PH via these or other mechanisms has yet to be fully elucidated.

YAP and TAZ are closely related transcriptional modifiers that have recently emerged as powerful effectors of mechanical signaling (Dupont et al., 2011; Halder et al., 2012; Aragona et al., 2013; Dupont, 2016). YAP/TAZ subcellular localization is tightly linked to ECM stiffness, with stiff matrix driving nuclear localization and activation, and soft matrix promoting cytoplasmic retention. Manipulation of YAP/TAZ levels can mimic changes in matrix stiffness with regard to proliferation, apoptosis, and cell differentiation, and can drive lung fibrosis (Dupont et al., 2011; Aragona et al., 2013; Liu et al., 2015).

Recent investigations into the complex signaling that regulates YAP/TAZ activity have only now begun to make this process clear. As members of the Hippo pathway, YAP and TAZ are biochemically regulated by phosphorylation by the serine-threonine kinases LATS1 and LATS2, with phosphorylation leading to cytoplasmic sequestration or degradation (Halder et al., 2012). However, a large variety of mechanical cues, including ECM rigidity, strain, shear stress, and adhesive area, facilitate YAP nuclear localization (Dupont et al., 2011; Wada et al., 2011; Aragona et al., 2013; Chaudhuri et al., 2016; Das et al., 2016; Nakajima et al., 2017) in a fashion potentially independent of YAP phosphorylation status (Dupont et al., 2011; Das et al., 2016; Elosegui-Artola et al., 2017). Inhibitor studies have demonstrated that this mechanical regulation requires cytoskeletal integrity (Dupont et al., 2011; Das et al., 2016), as well as actomyosin contractility (Dupont et al., 2011; Valon et al., 2017), and is inhibited by actin capping and severing proteins (Aragona et al., 2013).

YAP activation is also dependent on sufficient force-loading of integrins to allow talin unfolding and reinforcement of cell-matrix adhesions after integrin-ligand binding (Elosegui-Artola et al., 2016). Based upon these mechanical stimuli, it is not surprising that YAP nuclear localization is impaired by inhibition of FAK (Kim and Gumbiner, 2015; Lachowski et al., 2018), Src (Kim and Gumbiner, 2015), and Rho/ROCK signaling (Dupont et al., 2011; Yu et al., 2012). Most recently, two different reports demonstrated that YAP nuclear localization requires intact stress and strain transmission to the nucleus itself through integrity of the LINC complex (Driscoll et al., 2015; Elosegui-Artola et al., 2017). Nuclear strain transfer is notably dependent only on a patent actin cytoskeleton independent of microtubules and intermediate filaments, providing an explanation for the observation that YAP/TAZ nuclear localization is not impaired by microtubule depolymerization (Dupont et al., 2011). Elosegui-Artola et al. (2017) expanded on this finding by demonstrating that nuclear force application using AFM is sufficient to drive YAP nuclear translocation on soft matrix or stiff substrates with cytoskeletal disruption. Further examination using nuclear transport inhibitors and osmotic perturbations of nuclear shape demonstrated that rigidity-induced nuclear flattening led to increased cytosolic nuclear pore exposure and increased YAP nuclear import (Elosegui-Artola et al., 2017). This elegant series of experiments demonstrates that YAP and TAZ mechanotransduction is a direct readout of force transmission from the matrix through the actin cytoskeleton to the nucleus in the form of stress fiber-mediated nuclear flattening.

Upon nuclear translocation, YAP and TAZ associate with a number of promoter-specific transcription factors to drive mechanical signaling. The most notable and canonical of these factors are members of the transcriptional enhancer activator domain (TEAD) family, whose downstream products drive cellular proliferation and survival (Halder et al., 2012). However, there is mounting evidence for considerable cross-talk between YAP and TAZ and other key mechanotransduction pathways. Specifically, YAP and TAZ were found to be cofactors for SMAD signaling in the nucleus and involved in nuclear cross-talk with MRTFs (Varelas et al., 2008, 2010b; Speight et al., 2016; Yu et al., 2016; Kim et al., 2017), both of which have impacts on mechanical and TGF-β signaling. Additional YAP/TAZ interactions with both TGF-β/SMAD and β-catenin signaling are complex, involving some degree of mutual cooperativity in nuclear transcription but mutual sequestration and degradation in the cytoplasm (Varelas et al., 2010a; Heallen et al., 2011; Azzolin et al., 2012, 2014). In general, these cross-talk mechanisms appear to allow significant synergy in the setting of co-activation or co-inhibition while preventing pathologic feed-forward loops in the setting of mixed signaling.

Recent studies have revealed important roles for YAP and TAZ in vascular pathology and PH development. In vascular smooth muscle, YAP is induced after injury and promotes cellular migration, proliferation, and neointimal formation (Wang et al., 2012; Kimura et al., 2016). In PH, we and the Chan laboratory have found that YAP/TAZ upregulation in response to matrix stiffness promotes collagen deposition and cross-linking in PAAFs via miRNA-130/301 induction (Bertero et al., 2015). ECM remodeled by cells over-expressing YAP or miRNA-130/301 induced proliferation of PAECs, PASMCs, and PAAFs, demonstrating the potential for a positive feedback loop in response to ECM stiffening. Kudryashova et al. (2016) also identified an ECM-associated upregulation of YAP expression that was maintained by PASMCs derived from idiopathic PAH patients compared to controls. This fibronectin-associated elevation in YAP activity was linked to enhanced proliferation and survival via integrin signaling (Kudryashova et al., 2016). In PAECs, YAP/TAZ signaling is required for stiffness-associated increases in glutaminolysis; inhibition of either YAP/TAZ (via verteporfin) or glutaminolysis led to improvements in vascular stiffness, RV pressures, and RVH in the MCT rat model of PH (Bertero et al., 2016). Finally, we have recently examined PASMC response to matrix stiffening, and have found that YAP/TAZ signaling is a critical regulator of stiffness-associated remodeling behaviors (Dieffenbach et al., 2017). Mechanoactivation of YAP and TAZ promotes proliferation, LOX activity, enhanced contractility, and increased migration, all of which lead to further arterial stiffening and feedback amplification of remodeling. Mechanistically, this mechanobiological feedback is driven by YAP/TAZ-mediated suppression of COX-2 expression and prostaglandin signaling, which normally help maintain pulmonary vascular homeostasis (Dieffenbach et al., 2017). Genetic COX-2 deficiency alone in mice leads to PASMC remodeling and more severe hypoxia-induced PH (Fredenburgh et al., 2008, 2009), whereas early treatment with a long-acting prostacyclin analog can attenuate stiffness-induced remodeling behaviors and MCT-induced PH (Liu et al., 2016). Despite very different approaches to the investigation of pulmonary vascular remodeling, these studies each found a central role for YAP/TAZ activation in the process, highlighting the important role of mechanical signaling in PH development.

### Alternative Mechanical Effectors

Recent studies of the nuclear lamina have identified several key mechanosensing proteins that connect the nucleus to the actin cytoskeleton (now identified as the LINC complex) (Zhen et al., 2002; Padmakumar et al., 2004, 2005; Poh et al., 2012). Beyond their potential role in mechanical alterations in nuclear transport (as with YAP/TAZ, above) (Elosegui-Artola et al., 2017), these structural components can alter chromatin organization and impact gene transcription in response to substrate stiffness (Janmey et al., 2013; Swift et al., 2013; Alam et al., 2016). Genetic defects in these structural proteins lead to mesenchymal dysfunction and organ fibrosis, including cardiac fibrosis, but have not been linked to PH (Peng et al., 2006).

Like most significant cellular inputs, mechanical forces are likely to induce epigenetic changes in chromatin remodeling via DNA methylation and histone modifications; however, this process is relatively understudied. Some recent examinations have found that matrix stiffness and mechanical inputs can alter chromatin organization (Downing et al., 2013; Crowder et al., 2016; Le et al., 2016) and increase histone acetylation (Valenzuela-Fernandez et al., 2008; Li et al., 2011). These epigenetic changes can persist over time and through cell divisions, and may play a key role in long term changes in response to more transient alterations in the mechanical environment, a term called “molecular memory” (Yang C. et al., 2014; Heo et al., 2015). Further study of these mechanisms will allow a more complete understanding of mechanical signaling.

Finally, the rapidly expanding field of microRNA (miR) biology has led to the examination of miR regulation by mechanical signaling. A recent elegant investigation by Frith et al. (2018) screened for miRs upregulated by stiffness and/or RhoA signaling in mesenchymal stromal cells (MSCs). They verified two miRs (miR-100-5p and miR-143-3p) that biased MSCs further toward differentiation pathways associated with stiff ECM, demonstrating positive mechanobiological feedback via miR activity (Frith et al., 2018). In the endothelium, a large number of “mechano-miRs” have been found to respond to alterations in shear stress, and have been implicated in subsequent inflammatory signaling, apoptosis, and NO production (Kumar et al., 2014). In PH, we and the Chan group identified miR21, miR27a, and YAP/TAZ-dependent miR130/301 complex upregulation in response to ECM stiffness (Bertero et al., 2015). miR21 has been extensively studied in PH and found to have pleiotropic effects, with miR21 knockout mice displaying increased PH severity while miR21 inhibition leads to reduced vascular remodeling after hypoxia (Parikh et al., 2012; Yang S. et al., 2012; Iannone et al., 2014; White et al., 2014). Inhibition of the miR130/301 complex, however, decreased LOX production, fibrillar collagen deposition, and YAP nuclear translocation in cultured fibroblasts and the pulmonary vasculature *in vivo*, demonstrating disruption of mechanobiological feedback (Bertero et al., 2015). This translated to improved pulmonary pressures and RVH as well as decreased vascular stiffening in experimental PH models (Bertero et al., 2015).

Naturally, given the numerous miRs and their vast networks of targets, there are many miRs that have been found to modulate mechanical signaling pathways, including ECM components (Nanoudis et al., 2017), FAK (Eskildsen et al., 2011), β-catenin (Wu et al., 2016), and YAP (Xu et al., 2015). The role for these miRs in the regulation of mechanical signaling in PH, as well as their therapeutic potential, warrants further study (Negi and Chan, 2017). In particular, miRs are pleiotropic and often broadly expressed, so additional focus on the relative safety and efficacy of miR targeting of these mechanical pathways will be required.

## Concluding Remarks

Over the past several years, the increasing evidence for early development of PA stiffening and its contribution to RV workload in PH has led to an increased focus on arterial stiffness changes in the pathogenesis and potential early treatment of disease. Arterial stiffening has been found to occur fairly early throughout the pulmonary vasculature, affecting both large elastic arteries and small distal vessels. The downstream effects of this vascular stiffening include alterations in flow characteristics and changes in ECM composition that predispose to inflammation and continued pathologic vascular remodeling. The molecular mechanosensors that facilitate this process are now beginning to be understood, and their links to downstream signaling that locally reinforce matrix stiffening and force transmission delineated. Multiple recent studies have highlighted this process during PH development, and in so doing have demonstrated a potential therapeutic effect of disrupting this feedback loop. Further study of mechanobiological feedback in PH will be needed to more thoroughly interrogate these pathways and ultimately identify optimal targets for future therapy.

## Author Contributions

Review conception and design was by PD, LF, and DT. The figures were illustrated by MM. The manuscript was drafted by PD and LF and was approved by all authors.

## Conflict of Interest Statement

The authors declare that the research was conducted in the absence of any commercial or financial relationships that could be construed as a potential conflict of interest.

## Abbreviations

α-SMA: α-smooth muscle actin, an actin monomer upregulated in smooth muscle, myofibroblasts, and endothelial cells undergoing mesenchymal transition
AFM: atomic force microscopy – a method for measurements of local mechanical stiffness at the microscopic level
COX-2: Cyclooxygenase 2, the key enzyme for production of multiple prostaglandin lipid mediators
ECM: extracellular matrix
eNOS: endothelial nitric oxide synthase, a cellular enzyme responsible for the production of nitric oxide in endothelial cells
FAK: focal adhesion kinase, a mechanotransducer upregulated by integrin signaling
GSK3β: Glycogen synthase kinase 3-β, the core member of the β-catenin destruction complex
kPa: Kilopascal, SI units for pressure and stiffness
LINC: linker of nucleoskeleton and cytoskeleton complex, a complex of proteins along the nuclear membrane that facilitates interactions between the nucleus and actin cytoskeleton
LOX: lysyl oxidase, a collagen cross-linking enzyme
LV: left ventricle
MCT: monocrotaline, a compound that induces PH in a rodent model
miRs: Micro-ribonucleic acid oligonucleotides that bind to messenger RNA and regulate gene expression
MMPs: matrix metalloproteinases, enzymes responsible for enzymatic cleavage of ECM components
mPAP: mean pulmonary artery pressure, the average pressure measured in the proximal pulmonary arteries during catheterization
MRI: magnetic resonance imaging
MRTFs: Myocardin-related transcription factors, transcription factors activated by actin polymerization and known to be important mechnotransducers and activators of serum response factor
NFκB: Nuclear factor-κB, a transcription factor involved in inflammatory and immune responses, as well as responses to flow stresses in endothelial cells
NO: Nitric oxide, an exogenously produced vasodilatory signaling molecule
PA: pulmonary artery
PAAFs: pulmonary artery adventitial fibroblasts
PAECs: pulmonary artery endothelial cells
PAH: pulmonary arterial hypertension, Group 1 PH specifically arising from pathology in the pulmonary arterial tree
PASMCs: pulmonary artery smooth muscle cells
PECAM: platelet endothelial cell adhesion molecule, a cell-cell adhesion molecule expressed on endothelial cells
PGF1α: Prostaglandin-F1α, an enzymatic product of COX-2 activity
PH: pulmonary hypertension
PVR: pulmonary vascular resistance, defined as the ratio between the fall in pressure across the pulmonary arterial tree and the cardiac output
Rho GTPases: the Rho family of guanine trinucleotide phosphate hydrolyzing enzymes, known regulators of actin dynamics. The three canonical members are Ras-homolog gene family, member a (RhoA), Ras-related C3 botulinum toxin substrate 1 (Rac1), and Cell division control protein 42 homolog (CDC42)
ROCK: RhoA-associated kinase, a well-known downstream effector of RhoA
RV: right ventricle
RVH: right ventricular hypertrophy, enlargement of the RV free wall associated with PH progression
SMADs: similar to mothers against decapentaplegic, these intracellular proteins transduce signals from TGFβ superfamily members and act as transcription factors
SMC: smooth muscle cell
TAZ: Transcriptional co-activator with PDZ-binding motif, a transcriptional modifier and nuclear mechanotransducer
TEADs: TEA Domain proteins are the primary nuclear binding partners of YAP and TAZ
TG2: Transglutaminase-2, a calcium dependent cross-linking enzyme that remodels collagen I and other ECM components
TGFβ: A cytokine belonging to a superfamily of signaling proteins; TGFβ has frequently been associated with inflammation and fibrosis
TIMPs: tissue inhibitors of matrix metalloproteinases, secreted proteins that endogenously inhibit MMP activity
TRPs: Transient receptor potential, a group of ion channels located on the plasma membrane, some of which (TRPV4, TRPC3, TRPC6, TRPM7) are mechanosensitive
Wnts: Wingless/integrase, a family of signaling molecules and associated receptors
YAP: yes-associated protein, a transcriptional modifier and mechanotransducer
